# Secretome profiles of immortalized dental follicle cells using iTRAQ-based proteomic analysis

**DOI:** 10.1038/s41598-017-07467-3

**Published:** 2017-08-04

**Authors:** Lei Dou, Yan Wu, Qifang Yan, Jinhua Wang, Yan Zhang, Ping Ji

**Affiliations:** 1 Stomatological hospital of Chongqing medical university, Chongqing, China; 2 Chongqing key Laboratory for Oral Diseases and Biomedical Sciences, Chongqing, China; 3 Chongqing Municipal Key Laboratory of Oral Biomedical Engineering of Higher Education, Chongqing, China

## Abstract

Secretomes produced by mesenchymal stromal cells (MSCs) were considered to be therapeutic potential. However, harvesting enough primary MSCs from tissue was time-consuming and costly, which impeded the application of MSCs secretomes. This study was to immortalize MSCs and compare the secretomes profile of immortalized and original MSCs. Human dental follicle cells (DFCs) were isolated and immortalized using pMPH86. The secretome profile of immortalized DFCs (iDFCs) was investigated and compared using iTRAQ labeling combined with mass spectrometry (MS) quantitative proteomics. The MS data was analyzed using ProteinPilotTM software, and then bioinformatic analysis of identified proteins was done. A total of 2092 secreted proteins were detected in conditioned media of iDFCs. Compared with primary DFCs, 253 differently expressed proteins were found in iDFCs secretome (142 up-regulated and 111 down-regulated). Intensive bioinformatic analysis revealed that the majority of secreted proteins were involved in cellular process, metabolic process, biological regulation, cellular component organization or biogenesis, immune system process, developmental process, response to stimulus and signaling. Proteomic profile of cell secretome wasn’t largely affected after immortalization converted by this piggyBac immortalization system. The secretome of iDFCs may be a good candidate of primary DFCs for regenerative medicine.

## Introduction

A variety of factors secreted by MSCs (mesenchymal stromal cells) including growth factors, cytokines and chemokines is broadly defined as the MSC secretomes^1^. MSCs secretomes were considered to be therapeutic potential for regenerative medicine because of their angiogenic, trophic and immunomodulatory properties^2^. The effectiveness of MSCs secretomes on a series of diseases had been verified by many *in-vitro* or preclinical study^3–5^. Several types of MSCs could be isolated from dental tissue, including dental pulp cells (DPCs), stem cell from apical papilla (SCAP), dental follicle cells (DFCs), periodontal ligament cells (PLCs) and so on ref. 6. The secretomes of these dental tissue derived cells were acquired, and also showed good performance on tissue repair and neurodegenerative diseases in the previous studies^7–9^.

As a representative of dental tissue derived MSCs, dental follicle cells (DFCs) were isolated from dental follicle and had been proposed to have the capacity to differentiate into periodontium consisting of cementum, alveolar bone, and periodontal ligament^10^. Under certain conditions, DFCs can be induced to differentiate into chondrogenic, osteogenic, adipogenic and neurogenic cells^10^. DFCs secretomes was considered to be therapeutic potential. Conditioned medium (CM) from DFCs culture could induce osteogenic and cementogenic differentiation *in vitro* ^11, 12^ and the formation of dental pulp-dentin complex *in vivo* ^13^.

However, because the lifespan of primary MSCs and cell secretomes produced by MSCs is limited, a large number of MSCs need to be isolated from the tissues and expanded *in vitro*. Besides, this process was time-consuming and costly, which heavily impeded future clinical application of MSCs secretomes. So a population of immortalized MSCs which can secrete similar factors with original cells should be developped to satisfy the need of secretomes-based strategy.

In our previous study^14^, we immortalized human DFCs (iDFCs) using a piggyBac immortalization system. After immortalization, iDFCs maintained immunophenotype and differentiation capacity of DFCs, as well as high telomerase activity. However, the profile of iDFCs secretomes was unclear. Supposed that iDFCs secretomes were similar to (or not largely different from) original DFCs, they could be the excellent candidate for secretomes produced by primary DFCs. Thus, following our previous study, this study was to investigate the profile of iDFCs and DFCs secretomes using iTRAQ labeling combined with mass spectrometry quantitative proteomics.

## Results

### Cell morphology changed after immortalization

**Figure 1:**
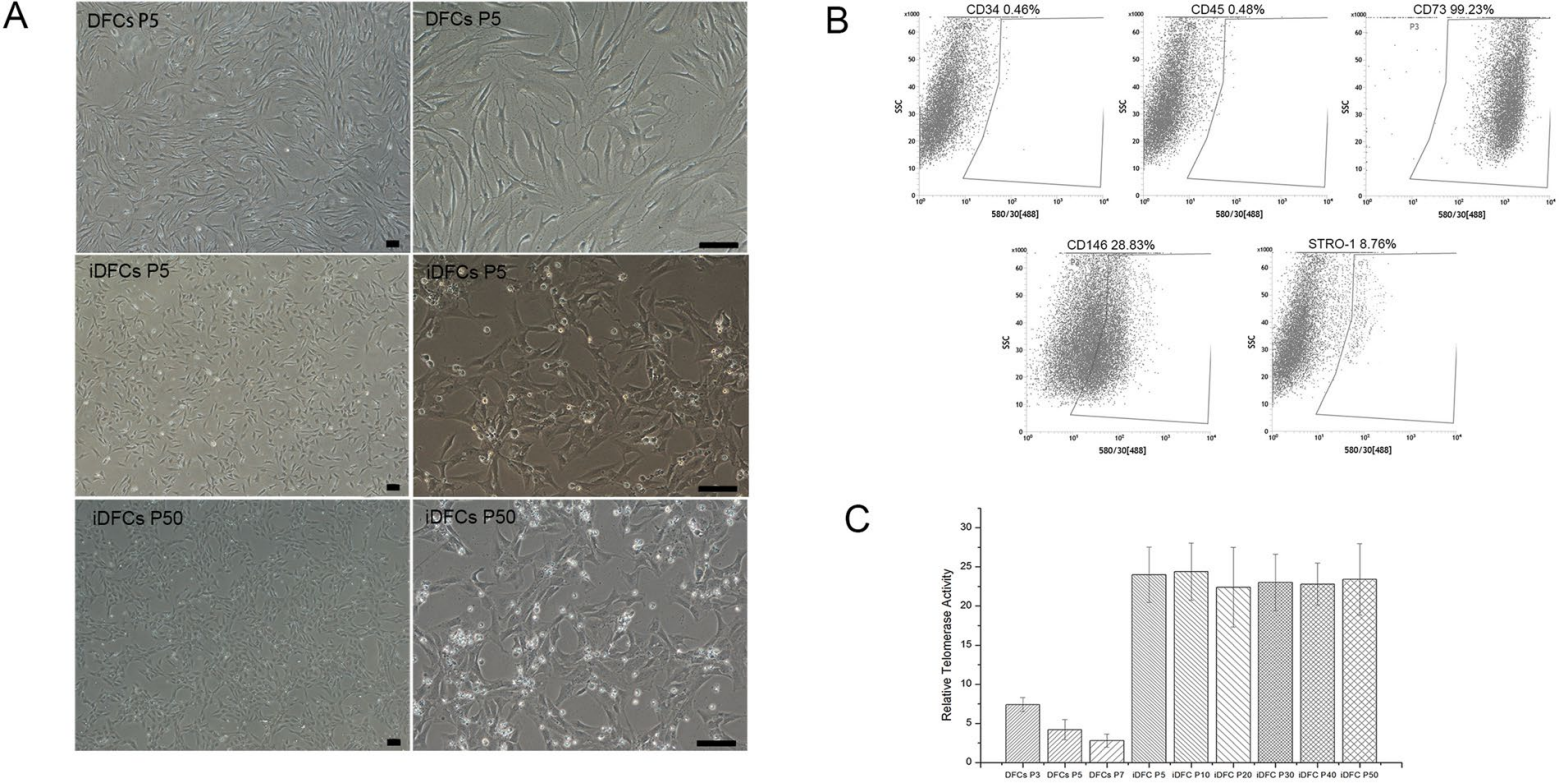
Morphology, phenotype and telomerase activity of immortalized DFCs. (**A**) The morphology of DFCs at passage 5 and iDFCs at passage 5, 50. Bar: 10 μm; (**B**) The phenotype of iDFCs; (**C**) The telomerase activity of DFCs and iDFCs at various passage. The telomerase activity of cells significantly increased after immortalization.

The original DFCs displayed fibroblast-like with a small size of cytoplasmic, while iDFCs lost typical fibroblast-like morphology and get shorter in length with relatively bigger nucleus (Fig. 1A).

### The phenotype and ability of multiple lineage differentiation

iDFCs displayed the phenotypes of CD73, CD90, and CD146 positivity and CD34, CD45 negativity. The percentage of STRO-1 positive cells in iDFCs are about 0.99% (Fig. 1B). The ability of multiple lineage differentiation was also confirmed by examining the potential for multilineage differentiation to adipocyte, chondrocyte, and osteocyte lineages.

### Telomerase activity enhanced after immortalization

Telomerase activity of iDFCs significantly increased compared with that of DFCs, and maintained at a high level till passage 50 (Fig. 1C).

### A large number of proteins were detected in iDFCs secretome

**Figure 2:**
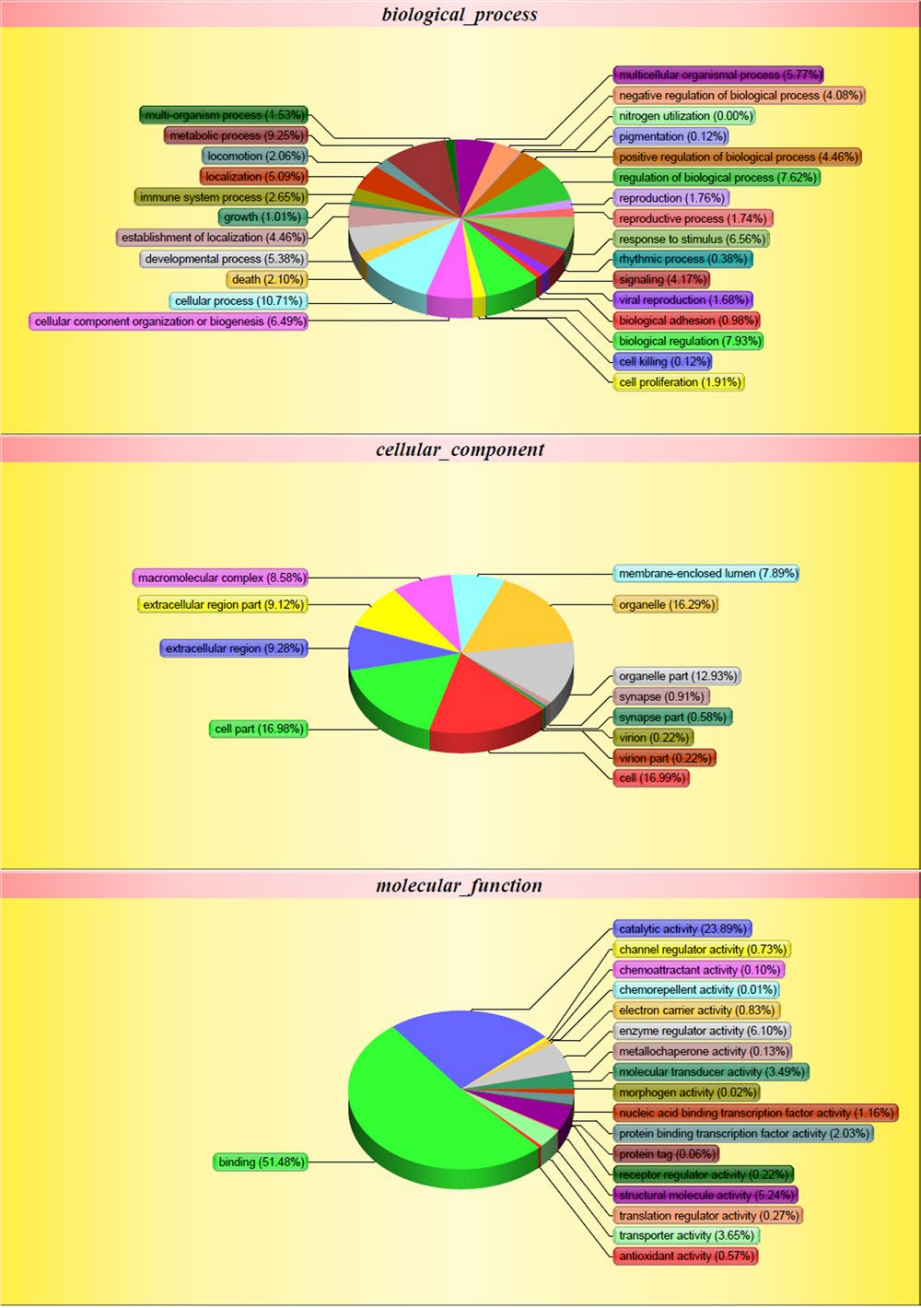
Gene Ontology term of identified protein in iDFC secretome.

**Figure 3:**
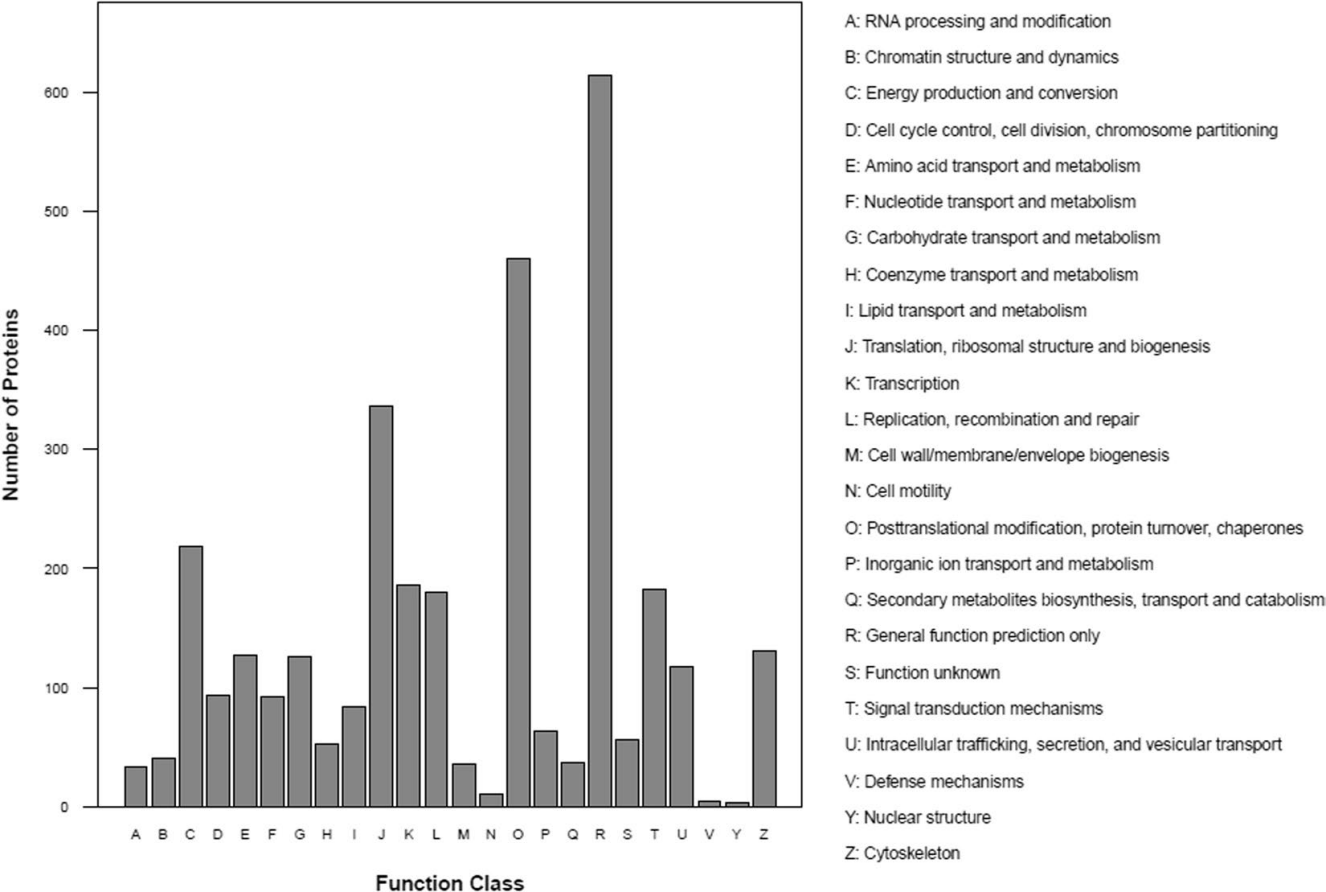
COG classification of all detected proteins in the CM of iDFCs.

A total of 2092 secreted proteins were detected in conditioned media of iDFCs. These detected proteins involved in cellular process, metabolic process, biological regulation, regulation of biological process, cellular component organization or biogenesis, immune system process, developmental process, response to stimulus, signaling, localization, multicellular organismal process, growth and so on. Gene Ontology (GO) term (molecular function, cellular component, biological process) of detected proteins in CM of iDFCs was shown in Fig. 2. COG (Clusters of Orthologous Groups of Proteins System classification) of all detected proteins in the iDFCs secretomes was shown in Fig. 3.

### Secretome profile was partly affected by immortalization

**Figure 4:**
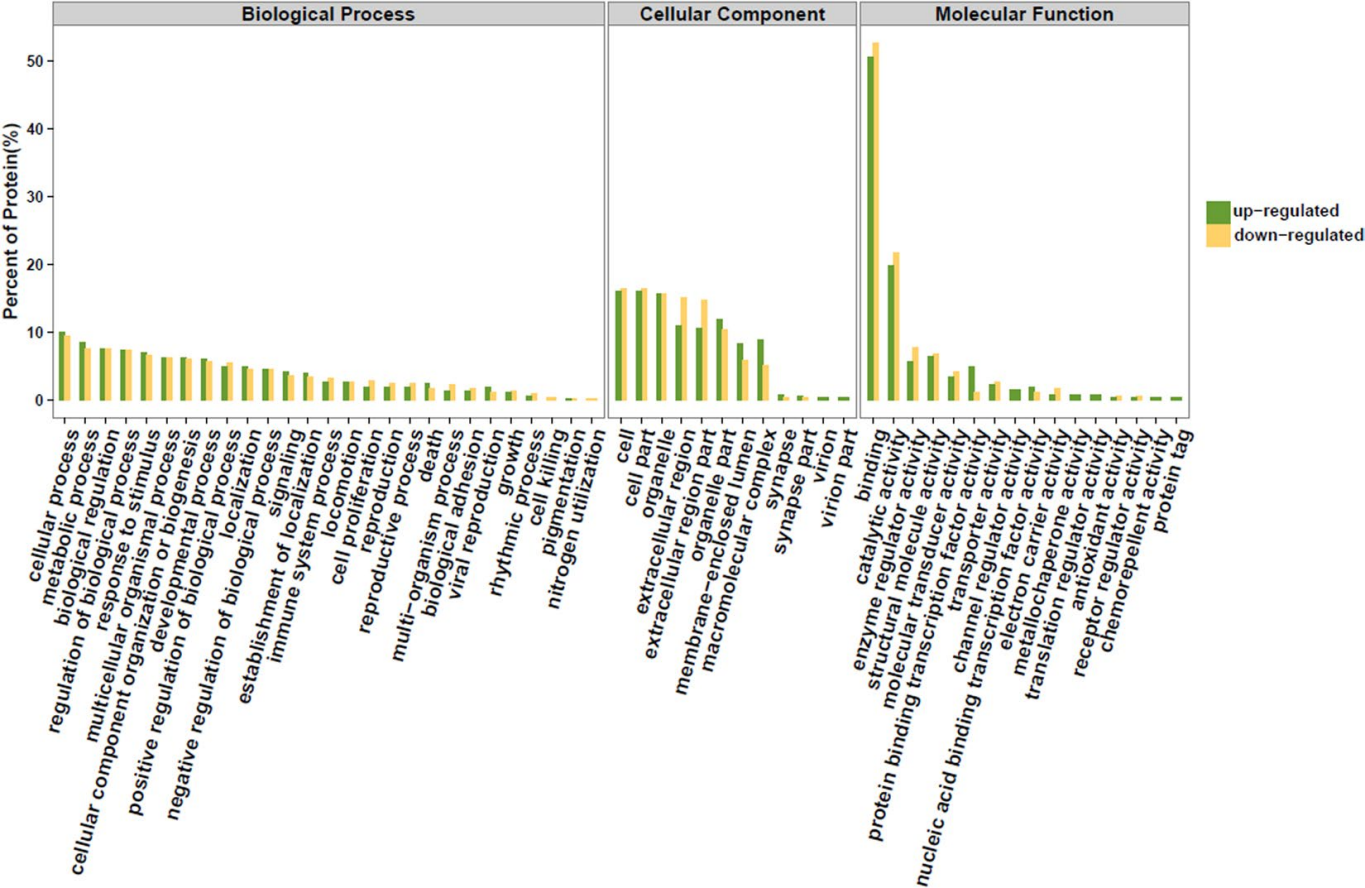
Gene Ontology term of up-regulated or down-regulated proteins in CM of iDFCs compared with CM of DFCs.

Only 12.1% of detected secretory proteins (253 proteins) were significantly affected by immortalization. Compared with primary DFCs, 142 protien up-regulated and 111 down-regulated in secretome of iDFCs (Supplementary file). The GO term of these differently expressed proteins was classified between the secretome of DFCs and iDFCs (Fig. 4). The pathway analysis of all up-regulated or down-regulated proteins after immortalization was shown in Supplementary file.

### DFCs or iDFCs secretome contained a series of bioactive factors

In the CM of DFCs or iDFCs, we detected collagen proteins (Type I, II, III, IV, V, VI, XI, XII), Nestin, MMPs (matrix metalloproteinase-2), TIMPs (metallopeptidase inhibitors), HSPs (heat shock proteins), PDGF (Platelet-derived growth factor), IGF-1,2(insulin-like growth factor-1 and -2), IGFBPs (insulin-like growth factor binding proteins), VEGF (vascular endothelial growth factor), bFGF (basic fibroblast growth factor) TGF-β1,2 (transforming growth factor beta 1, 2), HGF (hepatocyte growth factor) and SCF (stem cell factor), etc, which are closely related with proangiogensis, ECM remodeling, tissue repair and regeneration.

### Validation of selected differently secreted proteins

**Figure 5:**
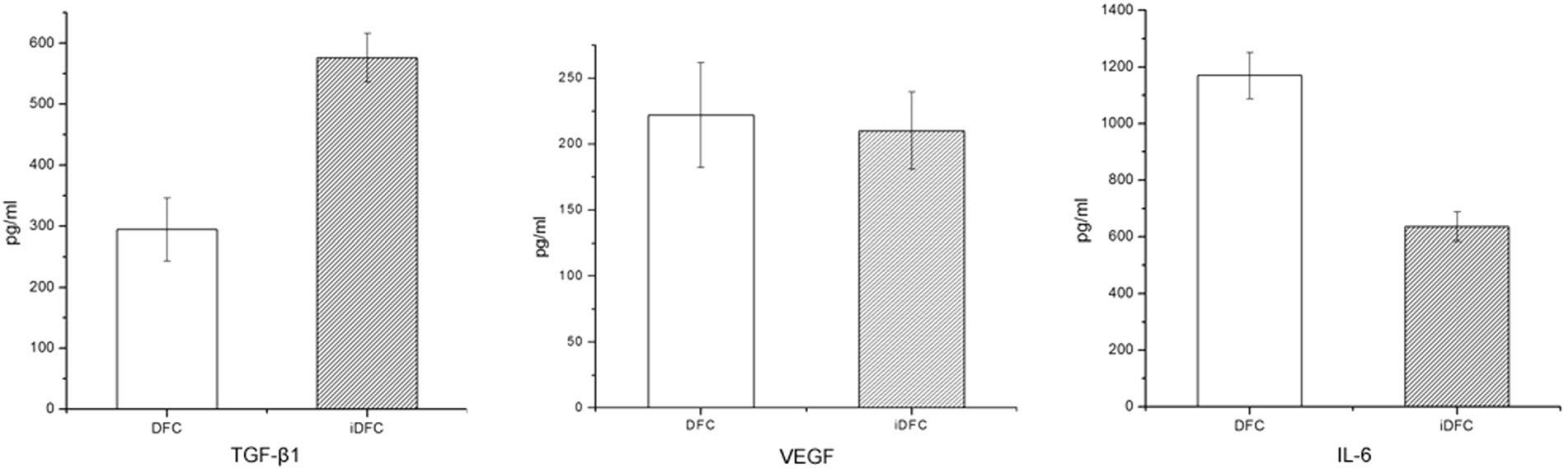
Concentration of cytokines in the CM of DFCs and iDFCs. Secretion of TGF-β1 increased in iDFCs compared to DFCs; Secretion of IL-1 decreased in iDFCs compared to DFCs; Secretion of VEGF didn’t significantly differ between DFCs and iDFCs.

Concentrations of selected secreted proteins were tested using Enzyme linked immunosorbent assay (ELISA). Compared with DFCs CM, the level of TGF-β1 up-regulated and the level of IL-6 (interleukin-6) down-regulated in CM of iDFCs. No significant difference in protein level (VEGF) was found between DFCs and iDFCs CM (Fig. 5). The ELISA results were consistent with the results of iTRAQ-labeling MS analysis.

## Discussion

DFCs are a population of cells isolated from dental follicle, which develops into periodontal tissues, such as periodontal ligament fibers, cementum and alveolar bone. DFCs have potential capacity of multi-differentiation, and are good choice for dental tissue engineering. However, like other types of MSCs, acquiring enough DFCs is also a major challenge before their application. Isolation of primary DFCs is costly and time-consuming, and limited lifespan of DFCs lead to the loss of their original character after several passages. To resolve this problem, this current study immortalized the primary DFCs using a piggyBac transposon-mediated system with overexpression of SV40 T-Ag. The results of our previous study^14^ showed multi-differentiation potential and immunophenotype of original DFCs could be retained based on this immortalization method.

MSCs secretomes were considered to be trophic, pro-angiogenic, immunomodulative, anti-inflammatory and anti-apoptosis, which largely contribute to the therapeutic potential of MSCs in regenerative medicine. The cell-free strategy based on MSCs secretomes attracted more attention and have demonstrated to be effective in many previous studies^3–5, 7–9^. Compared MSCs-based strategy, cell-free strategy is obviously an easier and safer way, which is less immunogenic and devoid of the risk of neoplasia. However, MSCs only produce a small amount of secretomes before they lose original character, so collecting enough MSCs secretomes for basic research or clinical therapy is also difficulty. To solve this problem, a large number of MSCs need to be isolated repeatedly from the tissues and amplified *in vitro*, which heavily impedes future clinical application of MSCs secretomes. Prolonging the lifespan and maintaining the character of original cells by immortalization could be a good choice. It was still unclear whether immortalization affect largly cell secretomes, which is closely associated with its further application. This present study for the first time comprehensively analyzed the secretome profile of a immortalized MSCs.

The results of this current study showed that immortalization partly changed the proteomic profile of DFCs secretome. Some secreted proteins were up-regulated, while some were down-regulated after immortalization using piggyBac system. Although the minority of secreted proteins (only 12.1%) were found to be differently expressed, the majority of the secreted proteins were not significantly affected by cell immortalization. Thus, the secretory function of DFCs was almost retained in the iDFCs. The secretomes of iDFCs may be a candidate for that of primary DFCs, which may be applied in regenerative medicine in future.

The iDFCs secretome included several key factors involved in tissue repair and regeneration such as collagens, MMPs, TGF-β, bFGF, SCF, and pro-angiogenic factors. Compared with primary DFCs, iDFCs secreted similar amount of angiogenic factors (VEGF, HGF, IGF-1, etc). These pro-angiogenic factors promote the proliferation and migration of endothelial cells, and maturation of newly-formed blood vessel^15^. Collagens and MMPs play a important role in ECM remodeling during tissue repair or regeneration^16^. SCF could induce homing and navigation of stem cells, which guarantee the participation of endogenous stem cells in the process of regeneration or repair^17^. bFGF can induce the proliferation of stem/progenitor cells and dentine formation^18–20^. TGF-β1 was closely associated with migration, proliferation and odontogenic/osteogenic differentiation of progenitor cells/stem cells, and stimulating matrix secretion^20–23^. Higher level of secreted TGF-β1 was found in immortalized cells, which implies immortalization may partly enhance the therapeutic potential of cell secretomes.

In this study, ElISA was done to verify the results of Mass spectrometry. Three proteins detected in MS analysis were selected. One of the three proteins (TGF-β1) up-regulated, one (IL-6) down-regulated and another (VEGF) didn’t change significantly after immortalization. The result of ELISA support the finding of iTRAQ-based MS analysis.

The piggyBac transposon system is adopted as the tool for immortalizing DFCs. This system can effectively catalyze integration and excision of transgenes in human cells between vectors and host genome through a direct “cut and paste” mechanism called transposition^24^. It is considered to be superior to other transposon system in different types of mammalian cells^25^. It has been reported that various MSCs derived from dental associated tissue were successfully immortalized using piggyBac transposon system^26, 27^.

This current study is subsequent to our previous study^14^. That previous study had evaluated the immunophenotype, multi-differentiation potential, telomerase activity, proliferation ability of iDFCs. For deep investigating iDFCs and evaluating the feasibility of therapy mode relying on iDFCs secretomes, this present study compared the profile of secretory factors before and after immortalization. As an important supplement, the results of this present study could provide information for further application of iDFCs.

Overall, our study provides a systemic secretome analysis of immortalized DFC revealing a number of secreted proteins which participate in various biological process. Immortalization using this piggyBac immortalization system didn’t largely change proteomic profile of cell secretome. The secretome of iDFCs could be a good candidate of original DFCs for regenerative medicine.

## Methods

### Isolation and culture of human dental follicle cells

All experimental protocols were approved by the Ethics Committee of Chongqing Medical University, and all methods were carried out in accordance with relevant guidelines and regulations. Isolation of human DFCs was done according to the method described in the previous study^26, 28^. Freshly extracted impact teeth for therapeutic pupose were collected in Chongqing stomatological hospitals in accordance with institutional ethical committee of this hospital. A written informed consent was acquired from each donner’ s parents. Dental follicle tissue was isolated from teeth, washed repeatedly with phosphate-buffered saline (PBS; Hyclone, U.S.A). Subsequently, the tissue was cut into 1 mm^3^ pieces, dissociated enzymatically for 40 min at 37 °C with 3 mg/ml Type I collagenase (Gibco, USA) and 5 mg/mL Dispase (Roche, Switzerland) in PBS supplemented with 15% fetal bovine serum (FBS, Gibco, USA). The suspension was filtered through a 70 μm filter. Then was pelleted by centrifugation at 1000 rpm/min for 5 min. The pellet was resuspended with culture medium. Then all cells were cultured in DMEM/F12 medium (Hyclone, U.S.A) with the addition of 10% FBS, penicillin (100 U/ml) and streptomycin (100 μg/ml) in an atmosphere of 37 °C, 20% O2 and 5% CO_2_. When passaging cells, a mixture of 0.25% trypsin and 0.01% ethylenediaminetetraacetic acid (EDTA) was used.

### PiggyBac Mediated immortalized human DFCs

For setting up immortalized DFCs using the method described by Wu *et al*.^14^, DFCs at passage 3 were transducted with piggyBac vector pMPH86(Provided by Wang *et al*.^26^), and piggyBac transposase expression adenoviral vector AdpBase (Provided by Wang *et al*.^26^). Hygromycin B was used for selection for about 1 week to establish stable iDFCs pools. Aliquots of iDFCs and original DFCs were stored in liquid nitrogen tank.

### Flow cytometry

Immunophenotyping of iDFCs was conducted at passage 4. iDFCs (4 × 10^5^ cells) were washed and resuspended in stain buffer (PBS with 1% FBS), containing saturating concentrations (1:100 dilution) of the following conjugated mouse IgG^1,k^ anti-human monoclonal antibodies: CD34-PE, CD45-PE, CD73-PE, CD90-PE, CD146-PE (BD Biosciences, San Jose, CA) and STRO-1-FITC (Biolengend, San Diego, CA) for 1 h at 4 °C. Cell suspensions were washed twice and resuspended in Stain Buffer for analysis on a flow cytometer (FACS Calibur; BD Biosciences) using the CellQuest ProTMsoftware (BD Biosciences).

### Multiple lineage differentiation

Osteogenic differentiation. iDFCs at were seeded onto 12-well plates, grown to 70% confluence, and incubated in the differentiation medium containing 10 nM dexamethasone, 10 mM b-glycerophosphate, 50 mg/mL ascorbate phosphate, 10 nM 1, 25-dihydroxyvitamin D3, and 10% FBS for 5 weeks. Cultures were fixed in 60% isopropanol, and mineralization of extracellular matrix stained with 1% Alizarin Red S.

Adipogenic differentiation. iDFCs were seeded onto 12-well plates, grown to subconfluence, and incubated in the adipogenic medium containing 1 mM dexamethasone, 1 mg/mL insulin, 0.5 mM 3-isobutyl-1-methylxanthine, and 10% FBS for 6 weeks. Cells were fixed in 10% formalin for 60 min, washed with 70% ethanol, and lipid droplets were stained with 2% (w/v) Oil Red O reagent for 5 min and washed with water.

Chondrogenic differentiation. iDFCs were seeded onto 12-well plates, grown to 70% confluence, and incubated in the differentiation medium containing cultured with TGF-β3 (10 ng/mL) for 2 weeks in 5% CO_2_ at 37 °C, with a fresh medium change every 3–4 days. Chondrogenesis was perfomed by staining with safranin-O.

### Telomerase activity analysis

The telomerase activities of DFCs and iDFCs were examined using TeloTAGGG™ Telomerase PCR ELISAPLUS (Roche, USA). Briefly, telomeric repeats (TTAGGG) were added to the 3’-end of the P1-TS-primer, and the resulting products were amplified by PCR with the Internal standard. The obtained PCR products were divided into two vessels, followed by denaturation and hybridization with digoxigenin-labeled probes. Then the products were checked with an antibody against digoxigenin and the peroxidase substrate TMB. Cell extract treated under 85 °C for 10 min were used as negative controls.

### Preparation of cell secretomes

DFCs (Passage 4–6) and iDFCs (Passage 5) were seeded onto T75 culture flasks respectively at a concentration of 1 × 10^6^ cells/mL. When reaching 70% confluence, the cells were washed thoroughly 5 times with PBS to remove any serum residues and were re-fed with 12 ml serum free DMEM/F12 medium. After culturing the cells for 24 h, the CM was collected, centrifuged, filtered using 0.22 um syringe filters (Milipore, Germany). The CM was concentrated at 3000 × g with a 3KD Amicon® Ultra centrifugal filters (Milipore, Germany), and then vacuum frozen dried. The powder sample was stored at −80 °C until further experiments.

### Protein extraction

The powder was resuspended in 200 μl of tetraethylammonium bromide (TEAB) buffer, disrupted using ultrasonication, and centrifugated at 12000 rpm/min. The supernatant was collected, and 4 times volume of cold acetone with 10 mM DTT was used to precipitate proteins at −20 °C for about 2 h. After centrifugation at 12000 rpm/min for 20 min at 4 °C, the precipitates were collected and washed with 800 μl of cold acetone for two times. After centrifugation at 12000 rpm/min for 20 min at 4 °C, the supernatants were removed and the precipitates were dried and stored at −80 °C for later use. After resuspended in 200 μl of TEAB buffer, the protein concentration was measured using the Branford assay kit (Solarbio, Beijing, China).

### iTRAQ labeling and Peptide Fractionation

The protein samples were dissolved and reduced with tris-(2-carboxyethyl) phosphine, alkylated with methyl methanethiosulfonate, trypsin digested and labeled with iTRAQ Reagent-8 plex Multiplex Kit (AB Sciex U.K.) according to the manufacturer’s instructions. All of the labeled samples were mixed with equal amount (CM of DFCs: 113,114; CM of iDFCs: 115,116). Next, the labeled samples were fractionated using a high-performance liquid chromatography system (Thermo Dinoex Ultimate 3000 BioRS) equipped with a Durashell C18 (5 mm, 100 Å, 4.6 × 250 mm) column.

### Mass spectrometry analysis

LC-MS/MS analysis was performed on an AB SCIEX nanoLC-MS/MS (Triple TOF 5600 plus) system. Samples were chromatographed using a 120-min gradient from 2 to 35% (mobile phase A: 0.1% (v/v) FA, 2% (v/v) ACN; mobile phase B: 0.1% (v/v) FA, 90% (v/v) ACN after direct injection onto a 20 cm PicoFrit emitter (New Objective) packed to 20 cm with Magic C18 AQ 3-mm 200 Å stationary phase. MS1 spectra were collected in the range 360e1460 m/z for 250 ms. The 20 most intense precursors with charge state 2e5 were selected for fragmentation, and MS2 spectra were collected in the range 50e2000 m/z for 100 ms; precursor ions were excluded from reselection for 15 s.

### Bioinformatic analysis

The mass spectrometry data was analyzed using ProteinPilot^TM^ v4.5 (Applied Biosystems); peptide identifications were made using the Paragon algorithm searching against the UniProt human protein database. Only unique peptides whose confidence was more than 95% were contained in iTRAQ labeling quantification, and protein with the unused value more than 1.3 were considered for further analysis.

To determine the biological and functional properties of the identified proteins secreted by DFCs and iDFCs, the identified protein sequences were mapped with Gene Ontology Terms (http://geneontology.org/). For this, homology search was first performed for all the identified sequences with a localized NCBI blastp program against NCBI nr database. The e-value threshold was set to less than 1e-5, and the best hit for each query sequence was taken account for GO term matching. The GO term matching was performed with blast2go v4.5 pipeline^29^. COG System (http://www.ncbi.nlm.nih.gov/COG/) were employed for the functional annotation of genes from new genomes and for research into genome evolution. Pathway analysis specifying the relationships between the interacting molecules was made according to the KEGG database (http://www.kegg.jp/).

### ELISA

To confirm these differentially-expressed proteins, ELISA was performed for further verification. In total, 4 × 10^6^ DFCs or iDFCs were grown on 150-mm culture plates and incubated overnight. Cells were washed with PBS and cultured with serum-free DMEM/F12 media. After 24 h treatment, supernatants were collected and centrifuged to remove the debris. Levels of IL-6, TGF-β1 and VEGF were determined using Human IL-6 ELISA kit (Solarbio, China), Human TGF-beta1 Quantikine ELISA Kit and Human VEGF Quantikine ELISA Kit (R&D Systems).

### Statistical Analysis

Proteins with an average fold change larger than 1.5 (DFCs: iDFCs ratio >1.5 or <0.67) were considered to be significantly differentially expressed. All quantitative experiments were done in three independent experiment and the results were determined by three independent experiment. Data were showed as mean ± SD. Statistical significance was determined by student’s t test and a value of p < 0.05 was considered statistically significant.

## Electronic supplementary material

Supplementary information.
